# Relationship between Serum Total Cholesterol Level and Serum Biochemical Bone Turnover Markers in Healthy Pre- and Postmenopausal Women

**DOI:** 10.1155/2014/398397

**Published:** 2014-05-15

**Authors:** Tae-Dong Jeong, Woochang Lee, Sung-Eun Choi, Jae Seung Kim, Hong-Kyu Kim, Sung Jin Bae, Sail Chun, Won-Ki Min

**Affiliations:** ^1^Department of Laboratory Medicine, University of Ulsan College of Medicine and Asan Medical Center, 88 Olympic-ro 43-gil, Songpa-gu, Seoul 138-736, Republic of Korea; ^2^Department of Nuclear Medicine, University of Ulsan College of Medicine and Asan Medical Center, 88 Olympic-ro 43-gil, Songpa-gu, Seoul 138-736, Republic of Korea; ^3^The Health Screening and Promotion Center, University of Ulsan College of Medicine and Asan Medical Center, 88 Olympic-ro 43-gil, Songpa-gu, Seoul 138-736, Republic of Korea

## Abstract

*Background*. The presence of common risk factors suggests that there is a relationship between osteoporosis and cardiovascular disease, possibly via dyslipidemia and inflammation. We investigated the relationships among the lipid profile, the inflammation marker high-sensitivity C-reactive protein (hsCRP), bone turnover markers, and bone mineral density (BMD) to assess the correlation between osteoporosis and cardiovascular disease and identify factors predicting osteoporosis. *Methods*. The study included 759 Korean women older than 20 years of age. The BMD, serum lipid profile, and levels of hsCRP, cross-linked C-terminal peptide (CTX), and osteocalcin were measured. We compared the serum biomarkers between groups with normal and low BMD and assessed the correlations between the levels of bone turnover markers and the lipid profile and hsCRP level. *Results*. The concentrations of CTX, osteocalcin, and total cholesterol were significantly higher in the low BMD group than in the normal BMD group in premenopausal women group. However, hsCRP was not correlated with these parameters. Multivariate logistic regression analysis revealed that TC (OR, 1.647; 95% CI, 1.190–2.279) and osteocalcin (OR, 1.044; 95% CI, 1.002–1.088) had an increased risk of low BMD in premenopausal women. *Conclusions*. These results indicate that total cholesterol concentration is correlated with the levels of bone turnover markers, suggesting that it might predict osteoporosis in premenopausal women.

## 1. Introduction

Osteoporosis and cardiovascular disease cause increased morbidity and mortality in elderly females. Several epidemiological studies have demonstrated that these two conditions are closely related [1–3], suggesting a possible link in the pathogenesis of osteoporosis and cardiovascular disease. The most plausible concept is that a common underlying mechanism triggers both osteoporosis and cardiovascular disease by affecting bone and blood vessels simultaneously. The most likely contributing factor is lipid levels.

A high total cholesterol (TC) concentration is related to the risk of cardiovascular disease. Lipid levels are also used to assess the risk of coronary heart disease, as cutoffs indicating that the commencement of treatment is appropriate and as goals in patient outcomes [4, 5]. In addition, high level of high-density lipoprotein (HDL) cholesterol is a protective factor for coronary heart disease, while an increased triglyceride level is an important component of metabolic syndrome [5]. It has been well documented that the atherogenic lipid profile is considered a key indicator reflecting the risk of cardiovascular disease [4, 5].

A possible association between lipid profile and osteoporosis has also been investigated. Some researchers have demonstrated that an atherogenic lipid profile is associated with a lower bone mineral density (BMD) [6–9]. However, other reports have found no relationship between the lipid profile and the BMD [10, 11].

Apart from the lipid levels, a possible role for inflammation has also been suggested in both conditions. Increased level of high-sensitivity C-reactive protein (hsCRP) is tightly correlated with an increased incidence of coronary heart disease in healthy individuals [12, 13]. Meanwhile, it has been reported that the relationship between hsCRP and osteoporosis suggests that a high hsCRP level is associated with low BMD [14].

In this study, we aim to investigate the association among lipid profiles, hsCRP, and bone turnover markers (BTMs) in healthy pre- and postmenopausal women to identify possible biomarkers that might predict osteoporosis.

## 2. Materials and Methods

### 2.1. Subjects 

The study population consisted of 759 Korean women older than 20 years of age who visited the Health Promotion Center of Asan Medical Center, Seoul, Korea, from January 2006 to December 2008, and in whom BMD and serum BTMs were measured. A self-administered questionnaire explored their medical, medication, and behavioral history. The height and weight of each subject were measured, while the subjects were dressed in light clothing without shoes, and the body mass index (BMI, kg/m^2^) was calculated. Following an overnight fasting, venous blood was drawn from each subject for laboratory tests. Women were excluded if they had undergone a hysterectomy or if they had taken drugs such as HMG-CoA reductase, estrogen, or bisphosphonate, which could affect lipid levels and BMD.

### 2.2. Biochemical Measurements

The concentrations of TC, calcium, alkaline phosphatase, phosphorus, triglyceride, and HDL cholesterol were measured by colorimetric methods using a Toshiba 200FR automated analyzer (Toshiba Medical Systems, Tokyo, Japan). The serum hsCRP concentration was determined using the CRP immunoturbidimetric method (Roche Diagnostics, Basel, Switzerland) on a COBAS Integra 800 analyzer (Roche Diagnostics). The serum concentrations of CTX and osteocalcin were measured using a chemiluminescence immunoassay (Roche Diagnostics) on an Elecsys 2010 automated analyzer (Roche Diagnostics).

### 2.3. BMD Measurements 

The BMD (g/cm^2^) was measured at the nondominant femoral neck and the anterior-posterior lumbar spine (L1–L4) using dual energy X-ray absorptiometry (Prodigy Advance with ver. 11.4 software; GE Lunar, Madison, WI, USA). The* in vivo* precision of the machine was 0.60% for the femoral neck and 0.66% for the lumbar spine. Each T-score was calculated using inbuilt software, and a mean ± SD of BMD was established with reference to data for healthy young women from northeastern Asia. According to the World Health Organization (WHO) definitions, osteopenia was diagnosed in the range (–2.5 SD < T-score <–1.0 SD) and osteoporosis was considered present when the T-score was ≤–2.5 SD at any site.

### 2.4. Statistical Analysis 

The measurements for pre- and postmenopausal women were compared using Student's *t*-test, except for hsCRP level, which had a positively skewed distribution. The values of this parameter were compared using the Mann-Whitney *U*-test, and the data were logarithmically transformed for use in further analyses. The concentrations of biochemical markers and BTMs were compared between two groups with different BMD statuses (normal and low BMD) using Student's *t*-test. Univariate logistic regression analyses were performed and a backward stepwise multiple logistic regression analysis considering all variables was then conducted to assess the independent association of the BMD with other independent variables. Odds ratios (OR) and 95% confidence interval (CI) were calculated. All statistical analyses were performed using SPSS ver. 19 (SPSS, Chicago, IL, USA). *P* values < 0.05 were considered statistically significant.

## 3. Results

### 3.1. Baseline Characteristics

The characteristics of the study subjects were summarized in Table 1. The mean ages of the pre- and postmenopausal women were 43.6 ± 6.3 and 57.5 ± 6.7 years, respectively. All of the following were significantly higher in postmenopausal women: BMI, hsCRP, calcium, alkaline phosphatase, phosphorus, CTX, osteocalcin, TC, and triglyceride.

### 3.2. BTMs and Biochemical Markers by BMD Status

**Figure 1:**
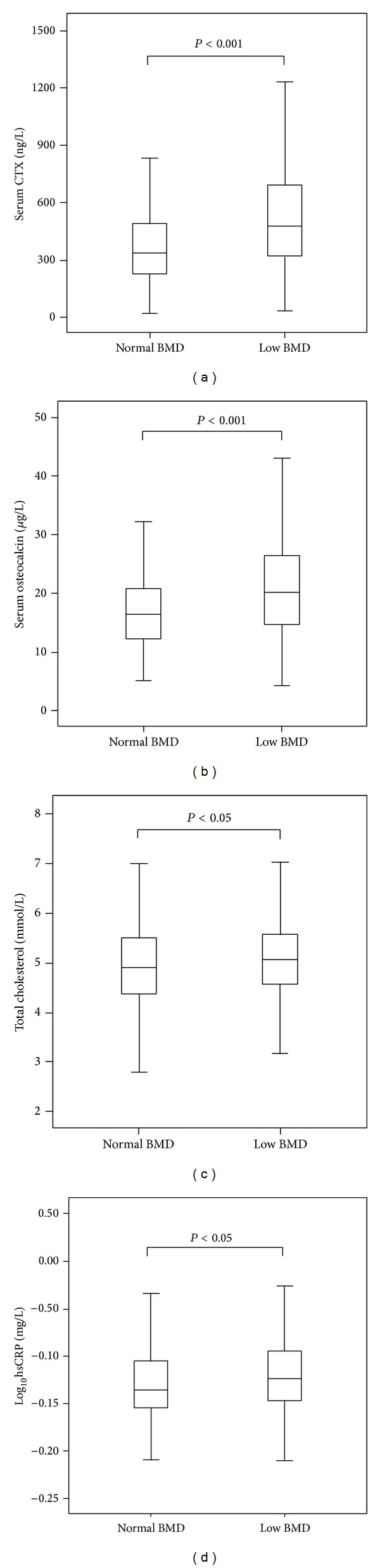
Serum CTX (a) osteocalcin, (b) total cholesterol, (c) log_10_hsCRP, and (d) concentrations in normal and low BMD subjects. The low BMD group was defined as those with osteopenia or osteoporosis as defined by the WHO classification. There were significant differences in the serum CTX (*P* < 0.001), osteocalcin (*P* < 0.001), and log_10_hsCRP (*P* < 0.05) levels between normal and low BMD groups. CTX, cross-linked C-terminal telopeptide; hsCRP, high sensitivity C-reactive protein.

We categorized subjects based on BMD status (using the WHO definition) into three groups: normal, with osteopenia, and with osteoporosis. Among the 759 studied subjects, 425 women were normal, 287 had osteopenia, and 47 had osteoporosis. Owing to the small number of subjects with osteoporosis, we combined the osteopenia and osteoporosis groups into the “low BMD” group to give a total of 334 subjects and compared the marker levels between normal and low BMD groups. The CTX and osteocalcin levels were significantly higher in low BMD group than that of normal group (both *P* < 0.001; Figure 1), as were TC and hsCRP (both *P* < 0.05; Figure 1), whereas triglycerides and HDL cholesterol did not differ significantly between these two groups.

**Figure 2:**
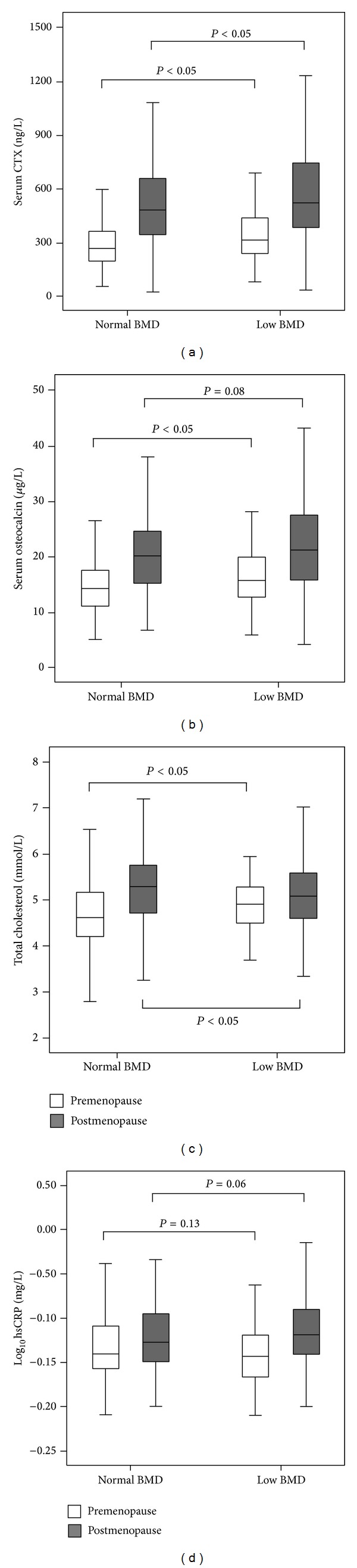
Serum CTX (a) osteocalcin, (b) total cholesterol, (c) log_10_hsCRP, and (d) concentrations in normal and low BMD subjects based on menopausal status. The low BMD group was defined as those with osteopenia or osteoporosis as defined by the WHO classification. CTX, cross-linked C-terminal telopeptide; hsCRP, high sensitivity C-reactive protein.

In a stratified analysis by menopausal status, both CTX and osteocalcin levels showed similar results (Figure 2). However, no statistical significance was found on hsCRP between normal and low BMD groups (Figure 2).

### 3.3. Stepwise Multivariate Logistic Regression Analysis

Multivariate logistic regression analysis revealed that the BMI (OR, 0.817; 95% CI, 0.756–0.884), TC (OR, 1.647; 95% CI, 1.190–2.279), and osteocalcin (OR, 1.044; 95% CI, 1.002–1.088) had an increased risk of low BMD in premenopausal women (Table 2). On the other hand, the age (OR, 1.094; 95% CI, 1.064–1.126), BMI (OR, 0.882; 95% CI, 0.826–0.942), and TC (OR, 0.649; 95% CI, 0.521–0.809) were statistically significant in postmenopausal women (Table 2).

## 4. Discussion

We found that the TC levels were significantly higher in the low BMD group compared to the normal BMD group in premenopausal women and were also positively correlated with the serum concentrations of CTX and osteocalcin. These results suggest that the pathogenesis of osteoporosis is related to cholesterol metabolism. An atherogenic lipid profile is thought to be associated with osteoporosis. It has been speculated that oxidized lipid is the common trigger of atherosclerosis and osteoporosis. Oxidized lipid stimulates atherosclerosis by promoting mineralization of the arterial wall and can cause osteoporosis by reducing bone mineralization and inhibiting osteoblast differentiation [15]. Our findings do support the previous research in premenopausal group. With our results, on the other hand, higher serum TC levels are associated with higher BMD in postmenopausal women. This result is quite opposite compared to the data of premenopausal group in our study; however, this finding is consistent with the previous study although its pathophysiology is still unclear [9].

Hormone-replacement therapy prevents cardiovascular disease by reducing the level of low-density lipoprotein (LDL) cholesterol and inhibits osteoporosis in postmenopausal women [16]. The use of statins is associated with an increase in BMD and a reduction in fracture risk, indicating that statins have anabolic effects on bone metabolism [17–20]. These findings provide further evidence of the relationship between lipid levels and osteoporosis.

The importance of other lipid markers and the levels of HDL cholesterol and triglyceride, with respect to BMD, has been debated [6, 9, 11, 21]. Here, we show that none of these parameters were correlated with biochemical markers of bone turnover and no differences in levels were noted between the two BMD groups, indicating that the serum levels of HDL cholesterol and triglyceride may not be directly related to bone metabolism.

Biochemical BTMs offer a dynamic measure of bone metabolism. BTMs arereleased into the circulation during bone formation or resorption and respond more rapidly and profoundly to changes in bone turnover than the BMD. In addition, BTMs are easily measured in the blood or urine and are increasingly assessed in clinical settings. Both osteocalcin and CTX are well documented, sensitive, and specific biomarkers for bone metabolism [22]. In particular, osteocalcin has several and complex biological functions. Osteocalcin plays a role in the regulation of bone mineralization and also regulates osteoblast and osteoclast activity [23]. In addition, osteocalcin was known to be related to energy metabolism [24].

The hsCRP concentration showed no correlations with BTM levels. hsCRP is the most sensitive marker for detecting subclinical inflammation, so this finding is inconsistent with the previous report [25]. Despite the absence of any perceived correlation, we cannot exclude a possible relationship between inflammation and osteoporosis. Several groups have provided evidence of a relationship between inflammation and osteoporosis. The incidence of osteoporosis is increased in individuals with inflammatory diseases [26–31]. It is possible that although hsCRP is a sensitive marker of inflammation, the dynamic hsCRP range in healthy individuals is too narrow to reflect changes in bone turnover. Therefore, despite a possible relationship between inflammation and osteoporosis, the hsCRP level might not be a useful marker for investigating bone metabolism or for predicting the development of osteoporosis.

Our study has several limitations. This is a cross-sectional analysis with no prospective follow-up of patients. And we have no information on the incidence of fracture and the ultimate clinical endpoint of osteoporosis. More well-controlled prospective studies would be needed to elucidate the relationship between candidate serum markers and the risk of osteoporosis.

In conclusion, our results indicate that a high TC level is associated with low BMD and atherosclerosis, suggesting that dyslipidemia is the common mechanism triggering osteoporosis and atherosclerosis in premenopausal women. However, the changes in the level of the inflammation marker hsCRP were not sufficiently prominent to be useful as a predictor of osteoporosis, despite the suggested association of inflammation with osteoporosis.

## Figures and Tables

**Table 1:** Clinical characteristics of the subjects based on menopausal status.

Variable	Premenopausal (*n* = 319)	Postmenopausal (*n* = 440)	*P* value
Age (years)	43.6 ± 6.3	57.5 ± 6.7	<0.001
Height (cm)	159.3 ± 5.4	156.2 ± 5.2	<0.001
Weight (kg)	56.0 ± 8.0	56.8 ± 7.3	NS
BMI (kg/m^2^)	22.1 ± 2.9	23.3 ± 2.9	<0.001
Spine *T*-score	0.17 ± 1.18	−0.93 ± 1.27	<0.001
Femur *T*-score	−0.02 ± 0.92	−0.68 ± 0.94	<0.001
Calcium (mmol/L)	2.26 ± 0.09	2.31 ± 0.08	<0.001
ALP (U/L)	50.1 ± 14.1	66.6 ± 21.8	<0.001
Phosphorus (mmol/L)	1.16 ± 0.16	1.24 ± 0.17	<0.001
Total cholesterol (mmol/L)	4.75 ± 0.78	5.15 ± 0.81	<0.001
Triglycerides (mmol/L)	1.03 ± 0.52	1.19 ± 0.59	<0.001
HDL-C (mmol/L)	1.64 ± 0.40	1.58 ± 0.38	<0.05
hsCRP (mg/L) (median and interquartile range)	0.39 (0.26–0.73)	0.59 (0.36–1.21)	<0.001*
CTX (*μ*g/L)	309 ± 172	546 ± 265	<0.001
Osteocalcin (*μ*g/L)	15.40 ± 6.00	21.69 ± 8.58	<0.001

ALP: alkaline phosphatase; BMI: body mass index; CTX: cross-linked C-terminal telopeptide; HDL-C: high density lipoprotein cholesterol; NS: not significant; TC: total cholesterol. Values are expressed as means ± standard deviations if not otherwise specified. *Analyzed using the Mann-Whitney *U*-test.

**Table 2:** Stepwise multiple logistic regression analysis to assess the association between bone mineral density as a dependent variable and other covariables based on menopausal status.

Variable	Odds ratio	95% confidential interval	*P* value
Premenopause			
BMI	0.817	0.756–0.884	0.001
TC	1.647	1.190–2.279	<0.05
Osteocalcin	1.044	1.002–1.088	<0.05
Postmenopause			
Age	1.094	1.064–1.126	<0.001
BMI	0.882	0.826–0.942	<0.001
TC	0.649	0.521–0.809	<0.001
CTX	1.001	1.000–1.002	<0.05

Analyzed independent variables: age, BMI, TC, hsCRP, CTX, and osteocalcin.

See Table 1.
